# Complement receptor 1 length polymorphism impacts glial functions relevant to Alzheimer’s disease

**DOI:** 10.1002/alz.087385

**Published:** 2025-01-03

**Authors:** Nikoleta Daskoulidou, Paul Morgan

**Affiliations:** ^1^ UK Dementia Research Institute at Cardiff University, Cardiff, South Glamorgan United Kingdom

## Abstract

**Background:**

Genome‐wide association studies (GWAS) in Alzheimer’s disease (AD) implicate complement in pathogenesis. Complement receptor 1 (CR1; CD35) is a top AD‐associated GWAS hit; the long variant, CR1*2, associates with risk. The roles of CR1 in brain and how variants influence AD risk are poorly understood. We investigated the impact of the AD‐associated CR1 length polymorphism on phagocytosis in induced pluripotent stem cell (iPSC)‐derived microglia and astrocytes.

**Methods:**

Donors were screened for erythrocyte expression of CR1 variants to identify homozygote donors (CR1*1/CR1*1, CR1*2/CR1*2); peripheral blood mononuclear cells (PBMCs) were isolated and reprogrammed to iPSCs. Clones were differentiated into microglia and astrocytes, confirmed by specific marker expression, then tested for phagocytosis of diverse targets (pHrodo‐E.coli bioparticles, pHrodo‐human synaptoneurosomes, Alexa‐488‐amyloid β fibrils), either unopsonised or opsonised with human serum or Factor I (FI)‐depleted human serum. Uptake of fluorescent targets was measured in real‐time.

**Results:**

CR1*1 and CR1*2 homozygote iPSC lines were established, differentiated into microglia and astrocytes, confirmed by cell‐specific marker expression. CR1 was expressed in all iPSC‐microglia and astrocytes for both variants. iPSC‐microglia and astrocytes were phagocytic and phagocytosis was enhanced by target opsonisation with human serum; FI depletion of serum reduced the enhancing effect. Comparison of CR1*1 and CR1*2 expressing microglia and astrocytes showed that expression of CR1*2, the AD risk variant, enhanced phagocytosis of opsonised targets.

**Conclusions:**

CR1 is an important component of microglial and astrocytic phagocytic activity; CR1*2‐expressing cells showed increased phagocytosis of opsonised targets, providing clues to mechanism of association with AD risk.

